# Non-invasive ventilation for acute hypoxemic respiratory failure, including COVID-19

**DOI:** 10.1016/j.jointm.2022.08.006

**Published:** 2022-10-22

**Authors:** Tommaso Rosà, Luca Salvatore Menga, Ambika Tejpal, Melania Cesarano, Teresa Michi, Michael C. Sklar, Domenico Luca Grieco

**Affiliations:** 1 Department of Emergency, Intensive Care Medicine and Anesthesia, Fondazione Policlinico Universitario Agostino Gemelli IRCCS, Rome 00168, Italy; 2 Istituto di Anestesiologiae Rianimazione, Università Cattolica del Sacro Cuore, Rome 00168, Italy; 3 Division of Cardiology, Department of Medicine, University of Toronto, Toronto ON M5S 1A1, Canada; 4 Interdepartmental Division of Critical Care Medicine, University of Toronto, Toronto ON M5S 1A1, Canada; 5 Department of Anesthesia and Pain Medicine, St. Michael's Hospital – Unity Health Toronto, University of Toronto, Toronto ON M5S 1A1, Canada

**Keywords:** Non-invasive ventilation, Hypoxemic respiratory failure, Self-inflicted lung injury

## Abstract

Optimal initial non-invasive management of acute hypoxemic respiratory failure (AHRF), of both coronavirus disease 2019 (COVID-19) and non-COVID-19 etiologies, has been the subject of significant discussion. Avoidance of endotracheal intubation reduces related complications, but maintenance of spontaneous breathing with intense respiratory effort may increase risks of patients’ self-inflicted lung injury, leading to delayed intubation and worse clinical outcomes. High-flow nasal oxygen is currently recommended as the optimal strategy for AHRF management for its simplicity and beneficial physiological effects. Non-invasive ventilation (NIV), delivered as either pressure support or continuous positive airway pressure via interfaces like face masks and helmets, can improve oxygenation and may be associated with reduced endotracheal intubation rates. However, treatment failure is common and associated with poor outcomes. Expertise and knowledge of the specific features of each interface are necessary to fully exploit their potential benefits and minimize risks. Strict clinical and physiological monitoring is necessary during any treatment to avoid delays in endotracheal intubation and protective ventilation. In this narrative review, we analyze the physiological benefits and risks of spontaneous breathing in AHRF, and the characteristics of tools for delivering NIV. The goal herein is to provide a contemporary, evidence-based overview of this highly relevant topic.

## Introduction

Optimal management of hypoxemic respiratory failure is highly debated. Avoidance of endotracheal intubation via non-invasive oxygenation strategies – high-flow nasal oxygen (HFNO), non-invasive ventilation (NIV), or continuous positive airway pressure (CPAP) – reduces risks of ventilator-induced lung injury and other serious complications (e.g., ventilator-associated pneumonia, diaphragmatic dysfunction, and delirium), improving clinical outcomes and quality of life even after hospital discharge.^[^[1], [2], [3]^]^ Conversely, patients with the greatest severity might require rapid escalation to invasive mechanical ventilation to avoid worsening outcomes in cases of delayed endotracheal intubation.^[^^4^^]^

For these reasons, the most recent guidelines are unable to provide definitive recommendations on the use of NIV for patients with acute hypoxemic respiratory failure (AHRF), suggesting the use of high-flow oxygen support over standard oxygen and NIV (although with very low certainty of evidence in the latter comparison) and caution in choosing among the available devices.^[^^5^^]^ However, neither these guidelines nor those by Rochwerg et al.^[^^6^^]^ considered patients with coronavirus disease 2019 (COVID-19).

Patients with AHRF have a dysregulated respiratory drive,^[^^7^^,^^8^^]^ which can generate high tidal volumes and promote patient self-induced lung injury (P-SILI).^[^^9^^,^^10^^]^ Strong inspiratory efforts and lung tissue inhomogeneities can produce injurious lung inflation patterns (i.e., the “pendelluft” phenomenon) that can further damage already inflamed lung parenchyma, leading to worsening clinical outcomes.^[^^11^^,^^12^^]^

Given the risk of P-SILI, the ideal respiratory support tool should not only improve oxygenation but also modulate respiratory drive and effort. This issue was of particular interest in critical care even prior to the COVID-19 pandemic, which further highlighted the topic's relevance, since an unprecedented number of patients are now affected by AHRF and treated in the most heterogeneous clinical scenarios. HFNO and NIV have been widely exploited in the current pandemic, with varying success rates.^[^^13^^]^ The enormous numbers of patients with COVID-19-associated respiratory failure admitted to intensive care units (ICUs) worldwide provided physicians and researchers an unprecedented volume of data, representing a unique opportunity to answer pressing questions that have evaded respiratory physiologists and intensivists for decades.

In this narrative review, we analyze the pathophysiology of spontaneous breathing in AHRF, the salient features of available non-invasive ventilatory strategies, and current evidence supporting the use of these modalities for managing AHRF in the context of COVID-19, highlighting lessons the last 2 years of pandemic have taught us and the remaining unresolved issues.

## Risks of Spontaneous Breathing

Aside from the benefits of avoiding mechanical ventilation (i.e., no sedation or muscle paralysis), maintenance of spontaneous breathing is advantageous for lung, heart, and diaphragm physiology. Indeed, spontaneous breathing helps prevent diaphragm dysfunction and atrophy,^[^^14^^,^^15^^]^ preserves cardiac preload and output,^[^^16^^,^^17^^]^ and yields increased aeration of the dependent lung (i.e., the dorsal and most atelectatic regions), minimizing ventilation/perfusion mismatch and improving blood oxygenation.^[^[18], [19], [20]^]^

However, critically ill patients with AHRF can have elevated respiratory drive due to combined factors including increased CO_2_ production and alveolar dead space, reduced pulmonary compliance, and enhanced central ventilatory response to CO_2_.^[^^7^^]^ This can shift the brain ventilatory curve toward a lower CO_2_, and the attempt to increase minute ventilation (i.e., increased respiratory rate and respiratory muscle activity) causes a stronger inspiratory effort.^[^^8^^]^ This translates into large swings in pleural pressure that generate high transpulmonary pressures and tidal volumes, potentially increasing stress on aerated lung tissue, which is markedly impacted by aeration loss from the disease process (i.e., “baby lung”).^[^^21^^,^^22^^]^

**Figure 1:**
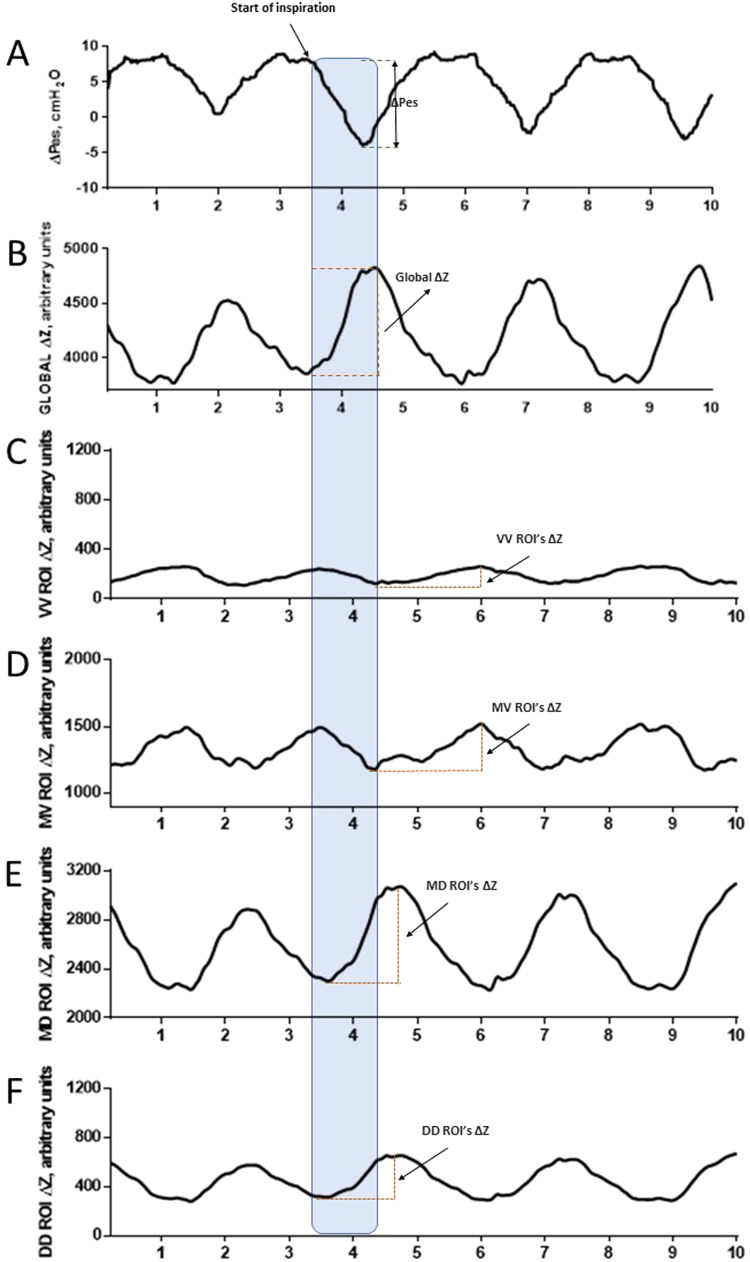
Pendelluft during spontaneous breathing. Representative patient tracings during spontaneous breathing, showing the pendelluft phenomenon between the ventral and dorsal lung regions. A: Esophageal pressure tracings showing the start of muscular inspiration corresponding to the point of esophageal pressure deflection and ΔPes representing inspiratory effort. B: Global ΔZ, expressed in arbitrary units, showing overall lung insufflation during inspiration. C–F: Ventral ROI ΔZ from C and D, demonstrating an initial emptying of these lung areas (blue rectangle), with air moving toward the dorsal ROI (E and F), which are instead characterized by an increased electrical impedance tomography signal. The opposite phenomenon occurs during expiration. The ΔZ of each ROI, corresponding to their relative tidal volume and occurring at different timepoints across respiratory cycles, are highlighted by the red dotted lines. DD: Dorso-dorsal; MD: Mid-dorsal; MV: Mid-ventral; ROI: Regions of interest; VV: Ventro-ventral.

Importantly, during spontaneous breathing, limiting transpulmonary pressure and tidal volume alone does not necessarily prevent harmful ventilatory patterns unless spontaneous effort is reduced.^[^^23^^]^ This illustrates that intense inspiratory effort might be dangerous *per se* and should thus be avoided. Intense swings in pleural and airway pressures promote recurrent alveolar openings and closings (i.e., atelectrauma^[^^24^^]^), while strong negative deflections in pleural pressure increase vascular transmural pressure and vessel permeability, favoring alveolar flooding and pulmonary edema.^[^^25^^]^ Moreover, negative pleural pressure swings are not uniformly transmitted throughout the lungs; some areas behave more like a “solid” (dependent, consolidated regions) and others more like a “fluid” (non-dependent, aerated regions) in terms of their mechanical response to distending stress. This inhomogeneous transmission of forces translates to a pleural pressure gradient, which generates an intra-tidal displacement of gas from non-dependent to dependent lung regions at early inspiration, a phenomenon called pendelluft (Figure 1).

Pendelluft can result in hidden movement of gas volumes, with regional overdistension of the dependent lung that cannot be detected by conventional tidal volume monitoring by a ventilator.^[^^12^^]^ Recently, a retrospective cohort study of 200 patients with AHRF showed that pendelluft, detected in 31% of those invasively ventilated, was associated with longer intensive care stay and fewer ventilator-free days among patients with a partial pressure of oxygen (PaO_2_) /fraction of inspired oxygen (FiO_2_) ratio <200 mmHg.^[^^11^^]^

In the diaphragm, strong inspiratory effort can cause muscle fiber inflammation, sarcolemmal rupture, and sarcomeric disarray, leading to diaphragm dysfunction and detrimental clinical outcomes.^[^[26], [27], [28]^]^ These physiological findings are supported by the association between persistently high inspiratory effort and non-invasive treatment failure.^[^[29], [30], [31]^]^ Cumulatively, these data warrant careful monitoring of respiratory effort by esophageal manometry,^[^^29^^,^^30^^]^ respiratory rate,^[^^32^^]^ and/or high tidal volumes.^[^[31], [32], [33]^]^ Delayed endotracheal intubation can worsen outcomes, especially in patients with severe hypoxemia (PaO_2_/FiO_2_ <200 mmHg).^[^^34^^,^^35^^]^

Non-invasive respiratory support can be administered safely and effectively in patients with a PaO_2_/FiO_2_ >200 mmHg. However, the optimal balance between benefits and harms of preserving spontaneous breathing with non-invasive respiratory support has yet to be fully elucidated for patients with a PaO_2_/FiO_2_ ≤200 mmHg. This is particularly important for patients affected by COVID-19, given the high failure rate of non-invasive respiratory support in this context and the shortage of equipment in such difficult circumstances.^[^^13^^]^ Advantages and disadvantages of the various ventilatory strategies are summarized in Table 1.

**Table 1:** Advantages and disadvantages of non-invasive respiratory support strategies.

Non-invasive strategy	Advantages	Disadvantages
HFNO	Simplicity of use Possible to deliver accurate FiO_2_ Small PEEP effect CO_2_ washout of upper airway Improved patient comfort Patient can speak, cough Possible to deliver treatment outside ICU	Only minor reduction in inspiratory effort Minor improvement of PaO_2_/FiO_2_ compared with CPAP and NIV
Face mask NIV	Improvement in oxygenation Reduction of inspiratory effort	Patient discomfort Air leaks common Risk of pressure ulcers Synchronization between patient and ventilator increases risk of high transpulmonary pressure and tidal volume
Helmet NIV	Possible to deliver higher PEEP Patient can speak, cough Improved dyspnea and oxygenation Reduction of inspiratory effort	Personnel training on interface necessary Inability to accurately monitor tidal volume
Helmet CPAP	Possible to deliver higher PEEP Patient can speak, cough Improved dyspnea and oxygenation	Personnel training on interface necessary Inability to accurately monitor tidal volume No effects on inspiratory effort in awake patient

CPAP: Continuous positive airway pressure; HFNO: High-flow nasal oxygen; NIV: Non-invasive ventilation; PEEP: Positive end-expiratory pressure; P-SILI: Patient self-induced lung injury.

## HFNO

The HFNO system allows delivery of heated, humidified flow up to 60 L/min at the desired FiO_2_ levels, through special nasal prongs.^[^^36^^]^ The gas flow source can vary, such as air/oxygen blenders, ventilators, and turbine flow generators, each allowing delivery of a high-flow air/oxygen mixture, matching the peak inspiratory flow and permitting precise FiO_2_ delivery across a wide range of respiratory rates and tidal volumes.^[^^37^^,^^38^^]^

HFNO allows development of a variable positive end-expiratory pressure (PEEP), which depends on the set flow, with higher PEEP developing when patients breathe with a closed (rather than open) mouth.^[^^39^^,^^40^^]^ Although small, these PEEP levels generate some alveolar recruitment, thereby improving oxygenation, as shown in both patients following cardiac surgery^[^^41^^]^ and those who are hypoxemic.^[^^42^^]^ Finally, although moderate,^[^^39^^,^^40^^,^^43^^]^ this flow-induced PEEP may be helpful in counterbalancing intrinsic PEEP in patients with dynamic hyperinflation, resulting in diminished work of breathing and improved comfort in patients with chronic obstructive pulmoriary disease.^[^^44^^]^

HFNO creates a washout effect of the upper airway dead space, particularly with flow rates >30 L/min.^[^^45^^,^^46^^]^ This mechanism leads to a reduction in partial pressure of carbon dioxide (PaCO_2_), especially in patients who are hypercapnic, modulates inspiratory effort in patients with AHRF, and provides passive oxygenation during procedural sedation.^[^^47^^,^^48^^]^

These cumulative physiological properties improve lung recruitment, with higher PaO_2_/FiO_2_ and reduced dynamic strain, improve thoracoabdominal synchrony,^[^^49^^]^ reduce respiratory rate, reduce minute ventilation, and improve patient comfort, with fewer displacements and oxygen desaturations than standard oxygen therapy.^[^^37^^,^^41^^,^^49^^,^^50^^]^ This strong pathophysiological rationale led to the FLORALI study, a randomized trial comparing HFNO, standard oxygen, and face mask NIV, which showed reduced mortality among patients receiving HFNO overall, and reduced intubation among the most severe HFNO-treated patients.^[^^51^^]^ These data showing efficacy, in combination with established safety, simplicity of use, and tolerability,^[^^37^^,^^45^^,^^50^^,^[52], [53], [54], [55], [56]^]^ make HFNO the first-line therapy for patients exhibiting AHRF in the most recent clinical guidelines.^[^^5^^]^

## NIV

NIV can be delivered through various interfaces – oronasal masks, face masks, and helmets – and with different ventilation modalities, usually pressure support ventilation (PSV) or CPAP.^[^^57^^]^ Each interface exhibits unique features, which can be exploited to optimize efficacy and tolerability. Traditionally, face masks – oronasal and full face – are used most often. The choice between mask type is based on patient comfort, face contour, and equipment availability as the difference in the internal dead space (which is higher in full-face masks) does not affect carbon dioxide (CO_2_) rebreathing, minute ventilation, inspiratory effort, or clinical outcomes.^[^^58^^]^ PEEP during face mask NIV and CPAP usually ranges from 5 cmH_2_O to 8 cmH_2_O; pressure support, when applied, is typically set from 7 cmH_2_O to 14 cmH_2_O. PSV-NIV increases airway pressure, improves arterial oxygenation, increases end-expiratory lung volume,^[^[59], [60], [61], [62]^]^ and augments cardiac function by reducing left ventricular afterload and right ventricular preload,^[^^63^^,^^64^^]^ ultimately decreasing inspiratory effort and work of breathing.^[^^60^^,^^65^^]^

Nevertheless, face mask NIV has some limitations. It is difficult to deliver higher PEEP because of air leaks, it produces skin necrosis with prolonged sessions, and patient discomfort can be an issue among those with hypoxemia as high PEEP is a suggested strategy for reducing inspiratory effort to improve outcomes.^[^^11^^,^^66^^]^

### Helmet NIV

The helmet is a transparent hood with a soft collar that contacts the body at the neck and/or shoulders but does not contact the patient's face. The size of the interface is determined by the patient's neck circumference. At least two ports are present, which are usually connected to separate tubing for inhaled and exhaled gas (i.e., a double-tube circuit). A standard Y-piece circuit can also be connected to a single port, although double-tube circuits are superior to Y-piece circuits in terms of ventilator pressurization performance and patient–ventilator interaction.^[^^57^^,^^67^^]^

The helmet Is a unique interface and therefore requires specific ventilatory settings for optimization. Helmets have their own compliance and must be well distended to guarantee the system's pressurization. The ventilator-delivered pressure must distend the compliant interface before it can pressurize the patient's airway, making inspiratory pressurization slower than face masks, with its speed inversely proportional to the helmet's baseline compliance. When this delay is too long, respiratory muscles may not be adequately unloaded, increasing work of breathing.^[^^68^^]^ For the same reason, system pressure decay after expiratory cycling is slower, increasing PEEP during expiration. To optimize the system performance, the following adjustments can be made.

#### High PEEP

Increasing PEEP (10–12 cmH_2_O) reduces helmet compliance, thus minimizing the amount of pressure support wasted on the interface and reducing airway pressurization time. In contrast to face masks, high PEEP reduces air leaks by forcing the helmet against the patient's shoulders for an optimized seal.^[^^69^^]^

#### Moderate high-pressure support

Similarly, since part of the pressure support is dissipated on the helmet surface, higher levels (10–18 cmH_2_O) can be used to adequately unload the respiratory muscles. Moreover, higher pressure support increases the washout flow, helping avoid CO_2_ rebreathing.

#### Fastest pressurization time

This setting optimizes the unloading of respiratory muscles during peak inspiratory effort.^[^^70^^]^

#### Gas conditioning

Gas conditioning, obtained either with heated humidifiers or heat and moisture exchangers (HME) to reach a minimum absolute humidity of 15 mgH_2_O/L,^[^^71^^]^ is recommended during face mask NIV.^[^^72^^,^^73^^]^ However, these settings cannot be generalized to the helmet interface. In one recent study, a double-tube circuit with no humidification allowed adequate conditioning of inspired gas, optimal comfort, and improved patient–ventilator interaction.^[^^74^^]^ The use of heated humidifiers or HME in this setting resulted in increased discomfort due to excessive heat and humidity in the interface, which was associated with more intense dyspnea.^[^^74^^]^ Humidification may be necessary when fresh gas flows >40 L/min are applied.^[^^75^^]^

#### Avoidance of CO_2_ rebreathing

The helmet has a volume around 18 L and behaves as a semi-closed mixing chamber with its own “helmet ventilation”. As such, part of the patient's exhaled gas is not eliminated from the helmet and mixes with gas coming from the inspiratory limb of the circuit.^[^[76], [77], [78]^]^ CO_2_ concentration inside the helmet depends on the relative balance between the patient's CO_2_ elimination and the system's washout flow (i.e., the helmet's minute ventilation).^[^^79^^]^ To limit this phenomenon, higher gas flows are necessary.^[^^80^^]^

Despite the use of optimal settings, asynchronies often occur during helmet ventilation; however, they usually do not affect its performance^[^^70^^,^^81^^]^ and may even exert a protective function since pressurization delay at inspiration causes uncoupling between the pleural pressure (patient effort) and airway pressure (pressure support), thus reducing positive transpulmonary pressure swings. Delays in expiratory cycling increase end-expiratory pressure, contributing to increase alveolar recruitment.^[^^29^^]^ Moreover, isometric inspiratory effort is not possible, even in cases of ineffective effort, due to the high-volume gas reservoir.

One important helmet limitation is that tidal and minute ventilation cannot be reliably monitored since a substantial portion of the inflation volume reflects distention of the helmet, rather than lung inflation. The “minute ventilation” displayed by the ventilator (and the flowby) represents the system's washout flow, rather than patient ventilation.

These cumulative helmet interface properties offer several advantages. First, patient comfort is improved compared with face mask NIV, in that it allows eating, coughing, and speaking, and avoids pressure ulcers.^[^^82^^]^ Improved tolerability allows helmet use for longer periods, even 48 continuous hours,^[^^83^^]^ minimizing the need for interruptions and the risk of NIV failure.^[^^84^^]^ Second, higher PEEP levels can be used with minimal leak or eye irritation.^[^^85^^]^ It is uncommon to reach PEEP levels higher than 5–8 cmH_2_O during face mask NIV,^[^^86^^]^ while levels of 12–15 cmH_2_O are easily achievable with a helmet.^[^^70^^,^[85], [86], [87]^]^ This represents a major physiologic advantage since higher PEEP may be crucial to minimize P-SILI and avoid endotracheal intubation in AHRF, particularly in patients with intense baseline inspiratory effort and more severe oxygenation impairment (PaO_2_/FiO_2_ ratio <150 mmHg).^[^[88], [89], [90]^]^

### Continuous positive airway pressure

Given the strong physiological rationale behind PEEP application during spontaneous breathing, CPAP has been proposed as an alternative to PSV-NIV in patients with hypoxemia.^[^[88], [89], [90], [91]^]^ In this context, CPAP has been regarded as a tool to increase end-expiratory lung volume without adding pressure support, which could increase the transpulmonary pressure and tidal volumes. It was proposed as the first-line treatment for *de novo* hypoxemic respiratory failure >20 years ago, in addition to treatment of cardiogenic pulmonary edema.^[^^6^^]^ With the COVID-19 pandemic, its application in patients with hypoxemic respiratory failure has become increasingly common.^[^^92^^]^ Traditionally, CPAP is administered through a high-flow generator (turbines, Venturi systems, or air/oxygen blenders) delivering fresh gas flow in an inlet port, and an adjustable PEEP valve connected to an outlet port. Its simplicity makes CPAP highly cost-effective in the emergency context and easily used outside of intensive care since ventilators are not strictly necessary. CPAP can be applied through face masks or helmets. When used with the helmet interface, flows of 35–40 L/min should be used to guarantee acceptable washout of the interface and avoid CO_2_ rebreathing.^[^^80^^]^ In the COVID-19 context, CPAP has become a common tool in standard and sub-intensive care. While this tool has helped the healthcare system face the pandemic emergency, it should be stressed that patients with hypoxemia who are treated with CPAP should be closely monitored by experts for signs of treatment failure. As described before, the face mask interface can be uncomfortable for long sessions when high levels of PEEP are applied, decreasing treatment adherence and causing significant air leaks. To overcome these limitations, helmet CPAP has been proposed.^[^^85^^,^^93^^,^^94^^]^

Indeed, helmet CPAP can rapidly improve the PaO_2_/FiO_2_ ratio of patients affected by AHRF, possibly via alveolar recruitment and decreased pulmonary shunt.^[^^95^^]^ On this basis, a small, randomized controlled trial demonstrated that compared with standard oxygen therapy, helmet CPAP reduced the intubation rate among patients with hypoxemia.^[^^96^^]^ In the 2000s, the first randomized controlled trial comparing CPAP delivered with face masks with standard oxygen therapy found no significant difference in endotracheal intubation rates likely because of participant heterogeneity, small sample size, and interface.^[^^97^^]^

CPAP was widely used at the outset of COVID-19, with conflicting results.^[^^92^^,^^98^^]^ Recently, a large adaptive, parallel group randomized clinical trial showed reduced intubation rates in patients treated with CPAP compared with a standard oxygen therapy group.^[^^99^^]^ Furthermore, Perkins et al.^[^^99^^]^ used the NIV ventilator module in the CPAP mode for almost 40% of their patients. While this may be a reasonable approach with the face mask interface, it should be avoided with the helmet since the latter has comparatively poorer performance in maintaining the desired PEEP level due to its higher system compliance.^[^^93^^]^ Thus, high continuous flows should be used, both to match the patient's peak inspiratory flow and to ensure adequate interface CO_2_ washout.^[^^80^^]^

### Patient monitoring during NIV

Patients treated with NIV must be carefully monitored and continuously assessed to identify early signs of treatment failure, to allow promptly proceeding to endotracheal intubation and ensuring protective ventilation.^[^^35^^]^ Worsening or lack of improvement in gas exchange, signs of respiratory muscle fatigue, feeling of unbearable dyspnea, development of respiratory acidosis, presence of unmanageable tracheal secretions, and hemodynamic instability are validated criteria for determining treatment failure; these are easily assessed at the bedside and have been used in clinical trials.^[^^51^^,^^83^^]^ In addition to absolute values, trends in these parameters over time may be even more valuable for correct patient assessment. Oxygenation improvement has also been associated with NIV success.^[^^80^^,^^100^^]^

Various predictive scores for non-invasive respiratory support have been developed in ongoing efforts to integrate different physiological parameters. The HACOR scale (based on heart rate, acidosis, consciousness, oxygenation, and respiratory rate) allows dynamic monitoring of intubation risk during face mask NIV.^[^^101^^]^ There are currently no validated scores for predicting helmet NIV failure, although *post hoc* analyses from a randomized clinical trial identified dyspnea score (assessed with a visual analog scale [VAS]) as predictive of treatment failure with both helmet NIV and HFNO.^[^^102^^]^

Finally, tidal volume and inspiratory effort are useful tools for guiding clinical decisions during NIV. An expired tidal volume >9–9.5 mL/kg PBW is a predictor of failure during face mask PSV NIV.^[^^31^^,^^33^^]^ Distinct from face masks, tidal volume monitoring during helmet PSV-NIV is impossible as the ventilator display value includes the gas volume needed to distend the interface, not that which reaches the patient's lungs. Monitoring esophageal pressure instead may help identify patients who will benefit from the support provided by both the helmet and face mask. One physiologic study showed that lack of reduced inspiratory effort over time is an early, accurate predictor of NIV failure.^[^^30^^]^ Strong inspiratory effort (>10 cmH_2_O) is one of the main determinants of P-SILI and may be the ideal monitoring tool during NIV. However, it requires esophageal balloon placement which, unfortunately, is not available in everyday practice. Despite its unreliability as an index of inspiratory effort,^[^^103^^]^ the respiratory rate is still commonly used as a surrogate of respiratory drive, with low or decreasing respiratory rates associated with successful non-invasive support.^[^^101^^,^^104^^]^

Monitoring remains paramount, and comprehensive patient evaluation that considers all of these factors should be performed routinely when using NIV as no clear-cut criteria are currently available to guide clinical decisions about whether – or when – to escalate treatment and/or proceed to endotracheal intubation.

## Summary of Current Evidence and Future Perspectives

Several randomized clinical trials have compared these interfaces in attempts to identify optimal non-invasive respiratory support parameters. Frat et al.^[^^51^^]^ compared standard oxygen with HFNO and face mask NIV sessions in patients with AHRF, demonstrating superiority of HFNO in terms of overall sample mortality and endotracheal intubation among patients with a PaO_2_/FiO_2_ <200. One year later, Patel et al.^[^^86^^]^ compared NIV delivered with a helmet or face mask, showing a significant reduction in intubation and mortality in the former group; importantly, helmet treatment was characterized by higher PEEP, longer continuous treatment, and decreased discomfort. These findings were confirmed by a recent meta-analysis by Ferreyro et al.^[^^105^^]^ who highlighted the potential superiority of helmet NIV compared with other interfaces in terms of endotracheal intubation rate.

Last year, the first direct comparison of helmet NIV and HFNO in patients with hypoxemia failed to detect any differences in respiratory support-free days after 28 days (the primary outcome) in patients with moderate-to-severe AHRF from COVID-19.^[^^83^^]^ Nevertheless, early, continuous treatment with helmet NIV with specific settings (PEEP 12 cmH_2_O and pressure support 10–12 cmH_2_O) reduced the rate of endotracheal intubation, increased the number of invasive mechanical ventilation-free days after 28 days, and improved oxygenation and dyspnea. No between-group mortality rate differences were detected.

Another recent randomized clinical trial (the RECOVERY-RS trial) compared CPAP, HFNO, and conventional oxygen on a composite of mortality and endotracheal intubation among patients with AHRF from COVID-19.^[^^99^^]^ CPAP treatment was superior to standard oxygen therapy, with endotracheal intubation driving the primary outcome difference. However, that study's limitations make interpreting the CPAP *vs*. HFNO comparison quite difficult. These limitations include wide inclusion criteria; inclusion of many patients treated outside the ICU, or who remained within the ward but without comprehensive monitoring; non-specified CPAP interfaces and settings; an adaptive design; substantial treatment crossover (17.1% of the overall sample, with 23.6% in the standard oxygen group); and lack of standardized intubation criteria.

Thus, to provide a clearer picture of this controversial topic, larger studies must directly compare the effects of non-invasive respiratory support tools on rate of endotracheal intubation and mortality, with careful patient selection.^[^^106^^,^^107^^]^

*Post hoc* analyses of some of the randomized controlled trials described previously have attempted to identify predictive variables for clinical guidance. A *post hoc* analysis of the HENIVOT trial showed that moderate-to-severe dyspnea, assessed with a VAS, was associated with increased endotracheal intubation rates, fewer respiratory support-free days, longer ICU and hospital stays, and higher in-ICU and in-hospital mortality; it thus constitutes an alarming sign.^[^^102^^]^ Moreover, in the same sample (comparing helmet NIV and HFNO), pretreatment PaCO_2_ <35 mmHg or PaO_2_/(FiO_2_ × VAS dyspnea) <30 (an index based on oxygenation impairment and dyspnea) identified a clinical phenotype (i.e., those with higher inspiratory effort) in whom helmet NIV produced the greatest clinical benefits.^[^^108^^]^ In other words, PaCO_2_ values and the PaO_2_/(FiO_2_ × VAS dyspnea) index during low-flow oxygen therapy differentiated patients who would especially benefit from initial treatment with helmet NIV from those in whom HFNO would instead suffice. While more research is needed to definitively end this controversy, progress is being made toward that goal.

## Conclusions

Various respiratory support tools are currently available for treating patients with AHRF. COVID-19-induced AHRF has put global healthcare systems under enormous stress, emphasizing the need for conclusive evidence regarding best practices among the available strategies in specific contexts. HFNO, NIV, and CPAP, with different interfaces, have been widely applied both before and during the pandemic, with variable success rates.^[^^13^^,^^105^^]^ These strategies have unique characteristics that should be understood and exploited toward optimizing treatment. Considering the unique features of each respiratory support tool, personalized treatments based on specific patient needs are ideal.^[^^106^^]^ Moreover, particular attention must be paid to patient monitoring, to promptly recognize signs of treatment failure and avoidingdelayed endotracheal intubation and protective ventilation.^[^^34^^,^^35^^]^

## Funding

This research did not receive any specific grant from funding agencies in the public, commercial, or not-for-profit sectors.

## Conflicts of Interest

Domenico Luca Grieco has received payments for travel expenses by Getinge and Air Liquide; speaking fees by Intersurgical, Gilead, Pfizer, General Electric Healthcare, and Fisher & Paykel; and a research grant by General Electric Healthcare. All other authors declare that they have no conflicts of interests.
